# Impact of single round of low dose CT lung cancer screening on cause of mortality in different socio-economic groups: a post-hoc analysis of long-term follow-up of the UKLS trial

**DOI:** 10.1016/j.lanepe.2024.100936

**Published:** 2024-05-20

**Authors:** Michael P.A. Davies, Daniel Vulkan, Rhian Gabe, Stephen W. Duffy, John K. Field

**Affiliations:** a Department of Molecular and Clinical Cancer Medicine Institute of Systems, Molecular & Integrative Biology, University of Liverpool, Liverpool, UK; b Centre for Evaluation and Methods, Wolfson Institute of Preventive Medicine, Queen Mary University of London, London, UK; c Centre for Cancer Screening, Prevention and Early Diagnosis, Wolfson Institute of Preventive Medicine, Queen Mary University of London, London, UK

**Keywords:** Lung cancer, CT screening, Mortality, Index of multiple deprivation

## Abstract

**Background:**

Lower socioeconomic status, as measured by the Index of Multiple Deprivation (IMD), is associated with higher rates of smoking-related disease mortality, and with poor uptake of cancer screening. Here we explore whether socioeconomic status impacts the effectiveness of a single round of low-dose-CT screening, or impacts other causes of death, in the UKLS LDCT screening trial.

**Methods:**

IMD quintiles were defined according to UK-wide data, with the deprived group defined as the lower two quintiles (Q1-2) and the less deprived as Q3-5. Follow-up data was obtained for lung cancer diagnosis (median follow-up 9.1 years) and cause of death (median follow-up 9.9 years). Outcomes were compared based on IMD group and trial arm (CT or control).

**Findings:**

More deprived quintiles were less likely to respond to the questionnaire, but this population was more likely to be selected for screening by the LLP risk model. Lower IMD quintiles benefitted from low-dose-CT screening in terms of lung cancer survival (HR 1.89, 95% CI 1.16–3.08) to the same extent as upper quintiles (HR 1.87, 95% CI 1.07–3.26). However, there was a bigger impact on deaths due to COPD and emphysema in more deprived quintiles.

**Interpretation:**

Whilst LDCT screening benefit for lung cancer was similar, significant impact on the rates of death from other smoking-related diseases, notably COPD and emphysema, was seen primarily in lower socioeconomic groups. Future research is required to confirm how lung cancer screening benefits other disease outcomes.

**Funding:**

10.13039/501100000664NIHR Health Technology Assessment Programme; NIHR Policy Research programme; 10.13039/100009855Roy Castle Lung Cancer Foundation.

**Research in context**

**Evidence before this study**

Whilst the benefits of low-dose CT early detection for those most at risk of lung cancer is now well-established, challenges remain for implementation of screening, including health disparity and equity, as some cancer screening programmes disproportionately benefit the least deprived socioeconomic groups. To investigate this, we conducted a comprehensive search on PubMed from the inception of the database until January 2024 using the search terms “lung cancer” AND “socioeconomic” AND (“CT” or “screening”). Most studies focussed on socioeconomic determinants of health and uptake disparities, and the limited data on socioeconomic impact on outcome differences was from the USA. We therefore set out to investigate outcome from a UK randomised control trial, with a focus on different socioeconomic groups.

**Added value of this study**

Unlike subsequent implementation studies in high prevalence, predominantly deprived, areas, the UKLS LDCT study recruited participants across the socioeconomic spectrum. This allows assessment of the impact of socioeconomic status on a variety of aspects, including: initial recruitment; selection for screening (using the LLP lung cancer risk model); lung cancer detection, stage shift, outcomes; and, long term mortality benefit from other diseases.

**Implications of all the available evidence**

The benefits of LDCT lung cancer screening in terms of improved lung cancer outcomes, from even a single round of screening, are comparable across different socioeconomic groups. Health equity is enhanced by use of risk profiling to select those for screening, as this favours application of LDCT in more deprived populations, where the need is greatest.

## Introduction

Deprivation, or lower socioeconomic status, as measured in the UK by the Index of Multiple Deprivation (IMD),^1^ is associated with numerous health challenges, including a high risk of lung cancer. There is a strong deprivation gradient for the proportion of cancer cases attributable to smoking in England,^2^ reflecting longstanding socioeconomic inequality in smoking prevalence^3^ with 23.8% living in the most deprived neighbourhoods current smokers in 2021, compared with 6.8% living in the least deprived neighbourhoods.^4^ Although itself associated with different smoking behaviours (heavier smoking in more deprived groups), socioeconomic status remains a risk factor for lung cancer after adjustment for smoking behaviour.^5^ Associations between socioeconomic differences and lung cancer outcome are more nuanced and weaker.^6^

In proposing lung cancer screening at the population level, both trials and implementation projects utilise risk assessment based partly on both age and smoking history to select at risk populations.^7^, ^8^, ^9^, ^10^ How the use of risk assessment in targeted lung cancer screening impacts its effectiveness in different socioeconomic strata, and how lung cancer screening might subsequently help abrogate, or worsen, health inequalities is important to address. This is especially true given that investment in cancer screening without accounting for the greater risk, and poorer outcomes, in more deprived sectors of society might increase disparities. For example, it has been reported that socioeconomic status has a detrimental influence on screening uptake for breast, cervix and colorectal cancers,^11^ but these screening programmes have broader selection criteria based on age and sex only.

The UKLS low-dose CT (LDCT) screening trial for lung cancer early detection has recently reported lung cancer mortality benefit for CT screening in a population selected as high risk for lung cancer.^9^ Here we explore the relationship between lung cancer risk and socioeconomic status and whether socioeconomic status impacts the effectiveness of LDCT screening on mortality outcomes in this high-risk trial cohort. The UKLS trial is particularly suited to this study as the target population had good representation of all IMD quintiles and after a single round of screening there is long-term follow-up for all-cause mortality, as well as lung cancer incidence.

## Methods

### Study design

This is a post-hoc analysis of the UKLS randomised trial using updated mortality data, stratified by IMD quintile (Q1 most deprived to Q5 least deprived) and whether participants received a low-dose CT scan (CT) or usual care (C, control). IMD score from the Office of National Statistics^1^ was provided anonymously based on postcode for the target population at the time of recruitment (2010). IMD scores were stratified into quintiles (Q1 most deprived to Q5 least deprived) based on UK-wide data. For outcome analysis, these were further grouped as more deprived (Q1-2) and less deprived (Q3-5). The IMD subgroups were defined in order analyse differences between IMD subgroups and also within subgroups based on LDCT status; with equal proportions of subjects receiving LDCT in each IMD group (chi-square 0.17, P = 0.90).

The UKLS trial has been described elsewhere^12^ and outcomes reported.^9^ Briefly, it was a randomised controlled trial, comparing LDCT screening with usual care using the “Wald Single-Screen” design in a population broadly representative of the UK at risk cohort.^12^ The study was based in two thoracic hospitals in the United Kingdom, the Liverpool Heart and Chest Hospital, on Merseyside (an area of relatively high deprivation), and Royal Papworth Hospital, in Cambridgeshire (an area of relatively less deprivation). Ethical approval was received from the Liverpool Central Research Ethics Committee (reference 10/H1005/74). Trial registration: International Standard Randomised Controlled Trial Register (reference 78513845).

A target population of 249,988 individuals aged 50–75 living in specific primary care trusts (PCTs) in the vicinity of the two hospital sites were invited by post to complete an LLPv2 risk model questionnaire^13^; 75,096 responders provided questionnaire data. A total of 4055 amongst those 8729 responders with the highest risk of lung cancer over the next 5 years (>4.5%) were selected for randomisation (excluding those: unable to give written informed consent; with any comorbidity which would unequivocally contraindicate either screening or treatment if lung cancer were to be detected; with a chest CT performed within the preceding year; or unable to lie flat). LDCT consisted of a single round of baseline LDCT, and follow-up LDCT at three to twelve months based on presence and size of indeterminate nodules (according to a standardised nodule management protocol^14^). Follow-up of nodules beyond the 12-month UKLS scan was at the discretion of participating centres.

### Outcomes

Outcomes from UK cancer and death registry data were provided by NHS Digital and the National Cancer Registration and Analysis Service (NCRAS) who were not aware of the participants’ allocated trial arm. Subject identifiers (NHS number) were provided to NHS Digital for those who consented to follow-up; outcome data was provided to the University of Liverpool at least twice per year from 2013 (following recruitment October 2011 to February 2013). The follow-up period for mortality (last death recorded) and for incidence of lung cancer (last diagnosis recorded) was up to September 2022 (data from: NCRAS to March 2018 and NHS Digital Cancer Registration and Mortality data to September 2022).

The primary outcome in this analysis was lung cancer mortality. This was defined as a death during the follow-up period where lung cancer was listed as the underlying cause of death in the UK civil registrations data provided by NHS Digital. Secondary outcomes were lung cancer incidence, mortality from causes other than lung cancer, and the distributions of the stage and histological type of the diagnosed cancers. Other underlying causes of death were analysed in the same way as lung cancer, grouping by ICD code into COPD and emphysema (ICD J43 & J44), cardiovascular disease (all ICD I codes), other cancers (ICD C, excluding C34), other lung diseases (e.g. pneumonia and other lung infections), other causes and unknown cause. Subjects were censored at date of last follow-up or at time of death from any cause other than the specific cause of death being tested. Stage and histology were provided by NCRAS or from case note review; for analysis early stage was defined as TNM stages I and II and late stage as stages III and IV.

### Statistical analysis

Outcomes were compared by Log–Rank test with associations compared by Kruskal–Wallis or Pearson's chi-square; hazard ratios were calculated by Cox proportional-hazard models. All statistical analysis was performed using IBM SPSS Statistics (version 26) or Stata, version 16.1.

### Role of the funding source

The UKLS was funded by the Health Technology Assessment programme of the National Institute for Health Research (NIHR). Michael Davies is a Roy Castle Lung Cancer Foundation Senior Research Fellow. Daniel Vulkan's and Stephen Duffy's contribution to this research was funded by the NIHR Policy Research Programme, conducted through the Policy Research Unit in Cancer Awareness, Screening and Early Diagnosis, PR-PRU-1217-21601. The funding source had no role in the design of our analyses, its interpretation, or the decision to submit the manuscript for publication. The views expressed are those of the author(s) and not necessarily those of the NIHR or the Department of Health and Social Care.

## Results

### Impact of recruitment and risk selection on IMD profile and risk factor profiles

**Fig. 1:**
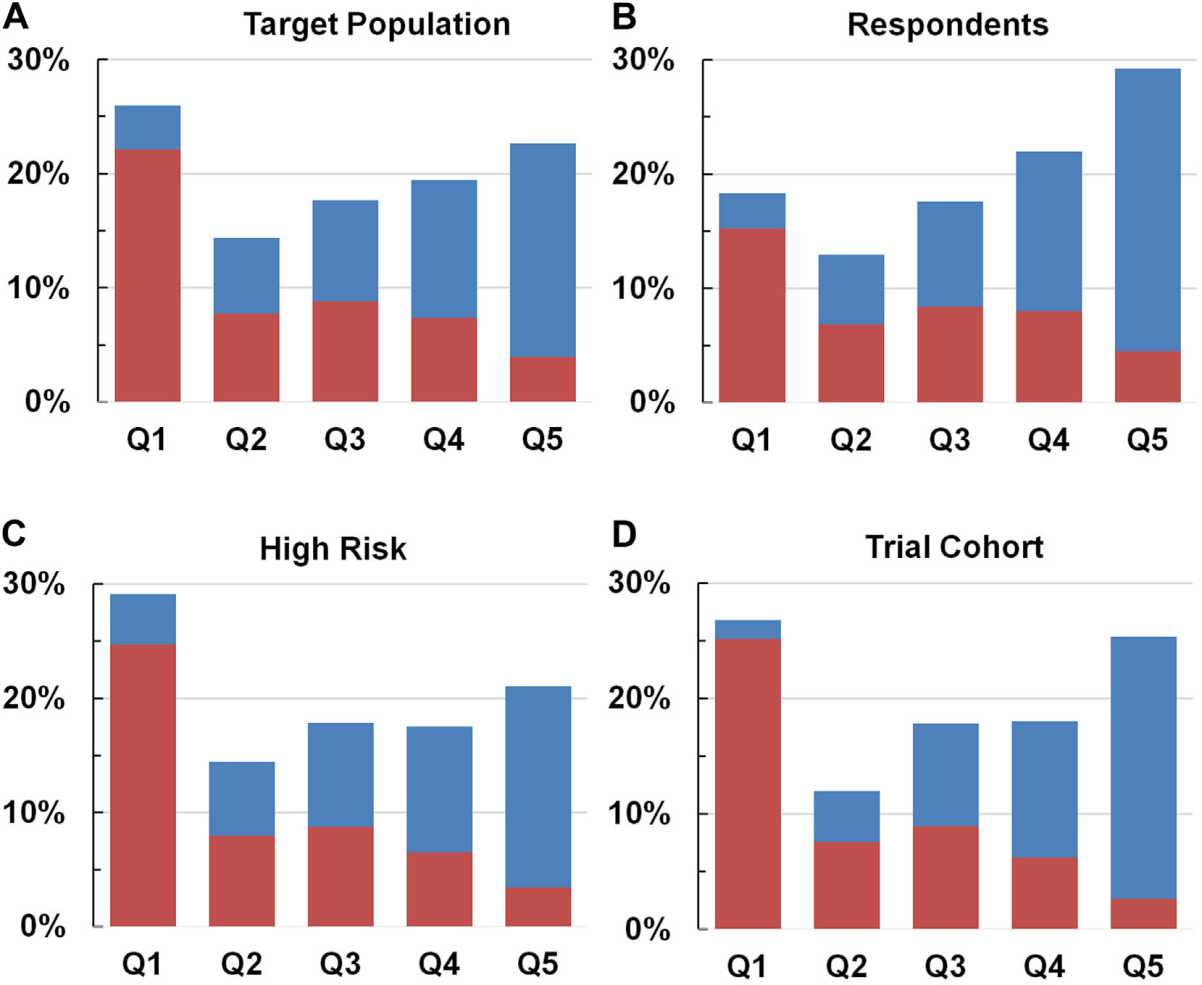
**IMD quintile and geographical distribution of UKLS target population (A), respondents (B), high-risk cohort (C) and trial cohort (D).** North/Liverpool = red, South/Cambridgeshire = blue.

The UKLS cohort was selected from two regions of England with differing IMD profiles, but which together had a reasonable spread of IMD by national quintiles (Fig. 1A), although notably with a bias towards the lower quintile (predominantly from the north–Liverpool) and upper quintile (predominantly from the south–Cambridgeshire). Amongst the questionnaire respondents (Fig. 1B) there is clear evidence of selection in favour of less deprived quintiles, partly explained by the greater response rates in the south (36.7% vs 26.1%). The IMD profile of the high-risk group eligible for LDCT screening (Fig. 1C) differed significantly from the respondents’ cohort and better reflected the total target population, with some enhancement of the lowest quintile. This resulted from application of the LLPv2 lung cancer risk score selection criteria (including age, sex, smoking duration, history of lung disease, asbestos exposure, personal history of lung cancer, and family history of cancer).^13^ During selection for trial participation (Fig. 1D) there was a minor shift in IMD profile, with a slight decreased representation of the lowest quintile and increase in the highest quintile. This was partly due to those in the most deprived quintile being less likely to respond to a secondary eligibility questionnaire, to attend clinic when invited or to consent (32.8% of those not recruited for those reasons being in IMD Q1), but also a higher proportion being ineligible for LDCT (32.7% IMD Q1, compared to 15.8–18% for other quintiles). However, both trial arms have the same IMD profile (chi-square P = 0.91, Supplementary Figure S1).

### Relationship between individual LLP risk score variables and IMD in UKLS responders

Selection effects for different risk factors were complex (see Supplementary Data). Most risk factors were more prevalent in more deprived quintiles across all respondents (Supplementary Table S1), but the distributions were different in the high-risk subjects following selection of those with a 5-year LLPv2 lung cancer >4.5% (Supplementary Table S2). In the trial cohort (Supplementary Table S3), only COPD and family history of lung cancer were significantly associated with deprivation, whilst pneumonia and personal history of cancer were associated with less deprived quintiles. Higher age was also statistically associated with less deprived quintiles in the high-risk cohort (Kruskal–Wallis asymptotic significances Q1 vs Q2 P = 0.015, Q1 vs Q3, Q4 or Q5 P < 0.001; Supplementary Figure S2B), albeit with only small absolute differences between the lowest (median 66, interquartile range 64–69) and highest IMD quintiles (median 68 y, interquartile range 65–71); related to the fact that to achieve a qualifying high-risk score, those in the less deprived quintiles had to be older, given that other risk factors were less prevalent. Males were over-represented in the high-risk cohort across all IMD quintiles, though less so in Q1 (Table 1, Supplementary Figure S2D). The proportion of heavy smokers was enhanced by risk-based selection across all quintiles (Supplementary Figure S2F), with Q1 having the greatest proportion of heavy smokers.

**Table 1:** Comparisons between IMD quintiles in UKLS trial participants.

	Q1	Q2	Q3	Q4	Q5	Q1-2	Q3-5
Subjects							
LDCT	525	235	352	342	500	760	1194
No CT	539	241	362	372	502	780	1236
Total	1064	476	714	714	1002	1540	2430
LLPv2 risk							
Median	7.7	7.4	7.2	6.8	7.1	7.4	7.1
(IQ range)	(5.6, 10.4)	(5.6, 10.4)	(5.5, 10.1)	(5.6, 9.8)	(5.5, 10)	(5.6, 10.4)	(5.6, 10.0)
P		1.00	0.86	1.00	0.028	0.003	
Age							
Median	67	68	68	68	68	67	68
(IQ range)	(65, 70)	(65, 70)	(65, 71)	(65, 71)	(65, 72)	(65, 70)	(65, 71)
P		0.31	0.002	<0.001	<0.001	<0.001	
Cig. Duration							
Median	47	44	42	41	40	46	41
(IQ range)	(38, 52)	(35, 50)	(31, 50)	(29, 49)	(27, 49)	(37, 51)	(30, 49)
P		0.001	<0.001	<0.001	<0.001	<0.001	
Cig. Pack Years							
Median	42	38	36	34	32	41	34
(IQ range)	(26, 56)	(24, 52)	(24, 50)	(18, 49)	(17, 45)	(25, 55)	(19, 48)
P		0.007	<0.001	<0.001	<0.001	<0.001	
All Smoking Duration							
Median	47	45	44	43	43	46	43
(IQ range)	(39, 52)	(35, 50)	(34, 50)	(33, 50)	(34, 50)	(38, 51)	(34, 50)
P		0.001	<0.001	<0.001	<0.001	<0.001	
Years since quit							
Median	2	7	8	9	9	3	9
(IQ range)	(0, 12)	(0, 17)	(0, 20)	(0, 21)	(0, 20)	(0, 14)	(0, 20)
P		<0.001	<0.001	<0.001	<0.001	<0.001	
Sex							
% male	67.5%	73.9%	75.4%	76.8%	81.1%	69.7	78.2
P		0.011	<0.001	<0.001	<0.001	<0.001	

Subjects numbers per group; Median and interquartile (IQ) range and P values: Kruskal-Wallace (KW) vs Q1 pairwise for each IMD quintile, Mann–Whitney U for Q1-2 vs Q3-5, or Chi-Square P values for difference to Q1 (significance values for two sided tests adjusted by the Bonferroni correction for multiple tests).

### Association of risk factors with IMD quintile in the trial population

Looking at the major risk factors (Table 1, Supplementary Table S3), these follow the patterns previously associated with IMD quintiles for smoking (greater duration and pack-years, plus less likely to have quit in the lower quintiles). The age of subjects in the upper quintiles (Q3-5) were significantly higher than for the lowest quintile, but there was no significant difference in LLP risk score except between the highest and lowest quintiles (Q1 and Q5). The actual differences in either age or smoking patterns, whilst significant, were small in real terms and did not significantly alter the risk profiles across IMD quintiles (Supplementary Figure S3A). Whilst ages tended to be higher (Supplementary Figure S3B) and smoking duration lower (Supplementary Figure S3C) in higher quintiles, the majority were aged between 65 and 70 and long-term smokers (>40 years) across all IMD quintiles, with no significant difference between trial arms.

### Differential impacts of LDCT on lung cancer incidence in IMD groups

The UKLS trial provides a useful insight into the role of deprivation in LDCT screening, as when grouping the lower two (Q1-2) and the top three quintiles (Q3-5) the expected association of IMD was clearly maintained for lung cancer incidence (Supplementary Figure S6A, Log Rank P = 5 × 10^−7^) and all-cause mortality (Supplementary Figure S6B, Log Rank P = 0.0045), when looking at the control group (limited to controls to avoid impact of LDCT detection). It should be noted, that in keeping with differences between the most deprived and other IMD quintiles for lung cancer risk factors (Table 1), all risk factors were significantly different between the IMD groups Q1-2 and Q3-5 (Table 1). The absolute differences were not large, but less deprived group was skewed towards lower risk, greater age, less smoking, longer time quit and more males.

**Fig. 2:**
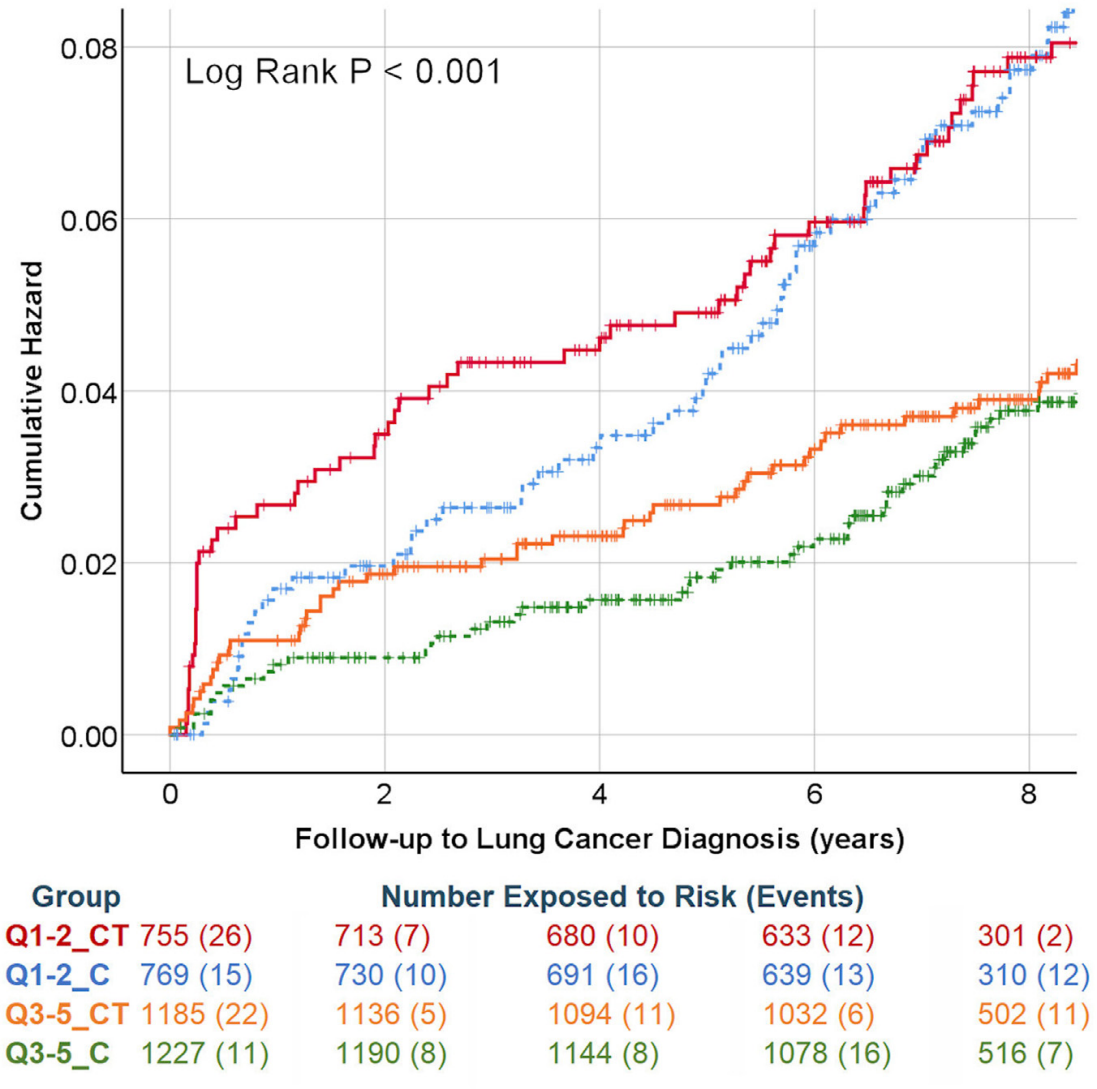
**Lung cancer incidence following UKLS intervention, stratified by LDCT and IMD**. Q1-2_CT = LDCT more deprived (red); Q1-2_C = Control more deprived (blue dotted); Q3-5_CT = LDCT less deprived (orange) Q3-5_C = Control less deprived (green dotted). The number exposed at time points 0, 2, 4, 6 and 8 years (B) are given, with the number of events in the subsequent time period (in brackets).

The median time to a lung cancer diagnosis was 4.7 years (n = 229) and median time to observation was 9.1 years (n = 3969); only 45 (1.1%) of the 3969 subjects who consented for follow-up were lost to follow-up (censored during study). UKLS Screen detected cancers were more likely to be in the lower IMD quintiles 22/1573 (1.4%) than upper ones 20/2482 (0.8%), in keeping with the overall higher rate of cancers in lower socioeconomic groups (Table 2). Notably, the increased diagnosis of lung cancers in the LDCT arm of the upper quintiles was maintained for almost 8 years, when cumulative hazards converged (Fig. 2), but the difference disappeared earlier, at 6 years, in more deprived quintiles.

**Table 2:** Lung Cancer incidence and outcomes by IMD group and trial arm.

Endpoint	IMD Quintile	Trial arm	Number of events	Cox hazard ratio (95% CI) significance	Deaths prevented/additional cancers diagnosed per 1000 subjects	Number of events	Cox hazard ratio (95% CI) significance
Lung cancer incidence	1–2	CT (N = 760)	50	1.024 (0.692–1.516) P = 0.904	1.6	100	0.690 (0.595–0.800) P < 0.001
		Control (N = 779)	50				
	3–5	CT (N = 1194)	41	1.187 (0.759–1.857) P = 0.453	5.2	77	
		Control (N = 1236)	36				
Lung cancer mortality	1–2	CT (N = 757)	24	0.806 (0.471–1.378) P = 0.430	7.0	54	0.641 (0.519–0.792) P < 0.001
		Control (N = 776)	30				
	3–5	CT (N = 1191)	15	0.736 (0.379–1.482) P = 0.365	4.4	36	
		Control (N = 1233)	21				
All-cause mortality	1–2	CT (N = 759)	131	0.839 (0.666–1.058) P = 0.139	28.9	288	0.895 (0.829–0.966) P = 0.005
		Control (N = 779)	157				
	3–5	CT (N = 1194)	182	0.969 (0.792–1.187) P = 0.764	3.8	375	
		Control (N = 1235)	193

The highest proportion of squamous cell carcinomas was seen in the most deprived quintile (35.6% of cancers), compared to 11.5–16.7% in other IMD quintiles (chi-square P = 0.005 for Q1 vs Q2-4, Supplementary Table S4). However, whilst also higher in the more deprived quintile (Q1-Q2 29.3%) than the less deprived (Q3-Q5 14.7%), this difference was not statistically significant.

### Differential impacts of LDCT on all-cause mortality and lung cancer mortality in IMD groups

**Fig. 3:**
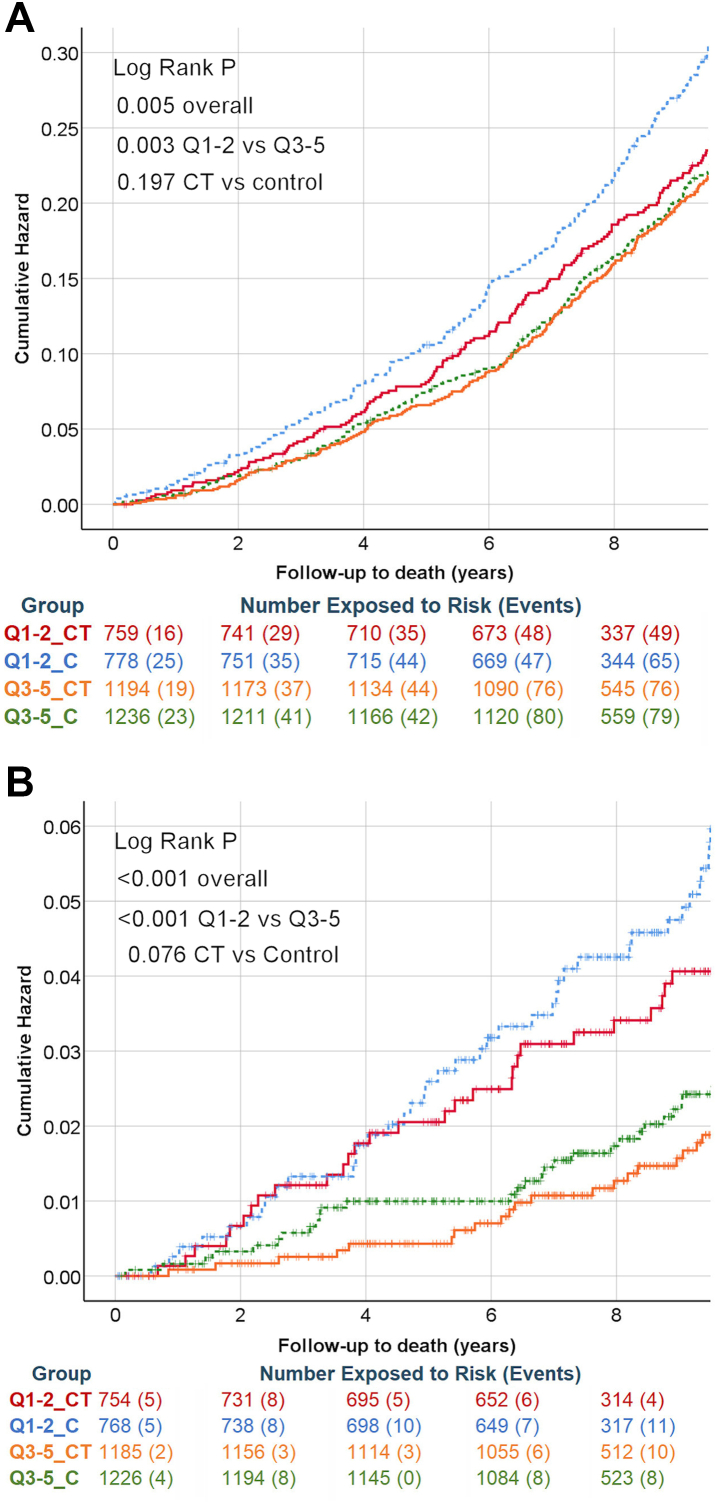
**All-cause mortality (A) and lung cancer specific mortality (B), stratified by LDCT and IMD.** Q1-2_CT = LDCT more deprived (red); Q1-2_C = Control more deprived (blue dotted); Q3-5_CT = LDCT less deprived (orange) Q3-5_C = Control less deprived (green dotted). The number exposed at time points 0, 2, 4, 6 and 8 years (B) are given, with the number of events in the subsequent time period (in brackets).

For mortality the median time to event was 6.5 years (n = 910) and median time to observation was 9.9 years (n = 3969). For all-cause mortality there was no observable benefit of LDCT for the upper IMD quintiles (Fig. 3A), whilst for the lower quintiles there was a significantly lower mortality (Log Rank P = 0.049) in the LDCT cohort.

Lung cancer specific mortality from time of screening was related to IMD and LDCT (Fig. 3B, Log Rank P < 0.001), and the pattern was somewhat different to all-cause mortality. The impact of less deprived IMD (log rank P < 0.001, HR 0.48 95% CI 0.35–0.68) was greater than the impact of LDCT (log rank P = 0.076, HR 0.74 95% CI 0.53–1.03). The benefit for the lower socioeconomic quintiles did not emerge until approximately 5 years after the LDCT screen (Log Rank P = 0.12 comparing lower quintile with and without LDCT). For the upper quintiles, there was an earlier difference in the rate of lung cancer deaths in the LDCT arm, primarily between three and five years of follow-up, but overall there was no significant difference (Log Rank P = 0.64 comparing upper quintile with and without LDCT).

Whilst trials arms (LDCT vs no LDCT) were balanced for all risk factors, not necessitating any adjustment to Cox regression analysis, the same is not true for IMD groups. As previously mentioned, there were small but significant differences in smoking duration (greater in Q1-2) and age (greater in Q3-5). We therefore included these within the Cox regression analysis and demonstrated that associations of IMD group with all cause morality, lung cancer mortality and COPD/Emphysema mortality were independent of age and smoking duration (Supplementary Table S5).

### Outcome from lung cancer by IMD group

**Fig. 4:**
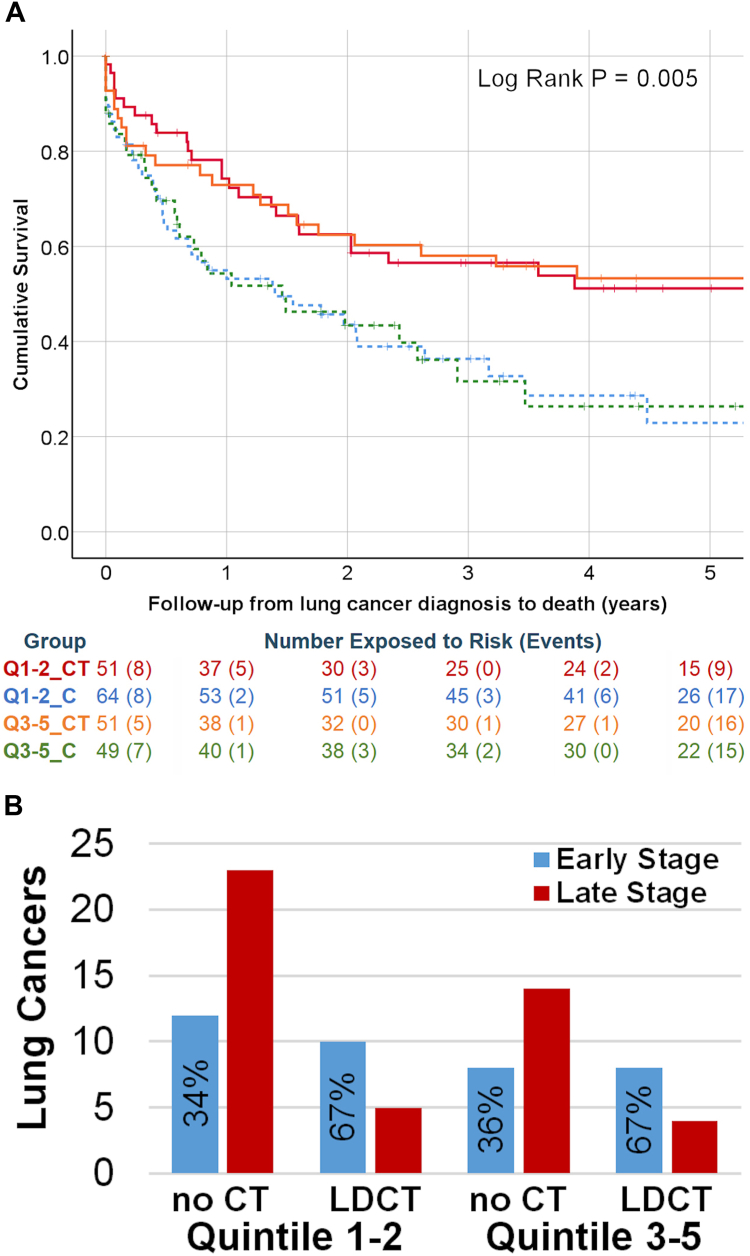
**Lung cancer outcome and stage distribution**. (A) Outcome following lung cancer diagnosis by LDCT group and IMD: Q1-2_CT = LDCT more deprived (red); Q1-2_C = Control more deprived (blue dotted); Q3-5_CT = LDCT less deprived (orange) Q3-5_C = Control less deprived (green dotted). The number exposed at time points 0, 1, 2, 3, 4 and 5 years (B) are given, with the number of events in the subsequent time period (in brackets). (B) Stage distribution of lung cancer, excluding screen-detected cancers: Early stage = IA-IIB, blue; late stage = IIIA-IV, red; % early stage.

For those with a lung cancer diagnosis, the median time to death was 0.58 years (n = 121), with a median time of observation of 1.4 years (n = 229). Outcome from lung cancer diagnosis (Fig. 4A) was not significantly different between IMD groups in the absence of LDCT screening, but, as shown previously, was significantly better for those in the LDCT arm of the trial.^9^ Lower IMD quintiles benefitted significantly from low-dose-CT screening in terms of survival from lung cancer [HR 1.89 (95% CI 1.16–3.08), log-rank P = 0.009] as did upper quintiles [HR 1.87 (95% CI 1.07–3.26), log-rank P = 0.023].

We investigated if stage shift may be responsible for long term benefit (Fig. 4B), i.e. for cancers detected subsequent to the LDCT screen (excluding the 42 screen-detected cancers). Even after screening ceased, there were more early-stage cancers detected in the LDCT group (chi-square 7.4, P = 0.007) and this was significant for lower quintiles (chi-square 4.5, P = 0.03), with the same pattern for the higher quintiles (chi-square 2.9, P = 0.09). No difference in the proportion of early-stage cancer was seen in terms of IMD quintile group (chi-square 0.08, P = 0.78).

### Impact of LDCT on other causes of death by IMD status

Other underlying causes of death were analysed in the same way as lung cancer (Supplementary Figure S7A). Whilst there was a lower proportion of lung cancer as a cause of death in both quintile groups with LDCT (−19% Q1-2 vs −20% Q3-5), there were fewer deaths due to COPD and emphysema in Q1-2 (−34%), but no reduction for Q3-5 (+4%). Time to death due to COPD or emphysema was notably shorter in the more deprived quintiles when no LDCT was performed (Supplementary Figure S7B, Log Rank P = 0.005), but with LDCT the rates were the same as for the higher quintiles, where death from COPD is less common.

Other lung diseases were also a less common underlying cause of death in those receiving LDCT only in lower quintiles (−32% Q1-2 vs +10% Q3-5), whilst unknown cause was less common with LDCT in both IMD groups (−34% Q1-2 vs −19% Q3-5). For cardiovascular disease the impact of LDCT in the upper quintiles was about half that seen for the lower quintiles (−30% Q1-2 vs −13% Q3-5). Unlike COPD, there was no significant difference in time to death from cardiovascular disease, although there was evidence of greater impact in the lower quintile (Supplementary Figure S7C).

There was a paradoxical increase in other cancers as a cause of death with LDCT in upper quintiles (+22%), that was not seen for lower quintiles (−5%). It should be noted that for the upper quintile group there were significantly more individuals for which personal history of cancer contributed to their lung cancer risk score and also that the average age was somewhat higher.

## Discussion

The UKLS study was based in two regions of the UK with differing socioeconomic profiles and in keeping with other screening studies participation was highest in those of higher socioeconomic standing (the less deprived). Despite this, inclusion of the LLP risk score for selection of trial participants resulted in a cohort with good representation of all IMD quintiles.

Based on improved survival from lung cancer following diagnosis, it is clear that there was a long-term benefit of LDCT screening following a single screen, related to detection at an earlier stage post-screening, irrespective of socio-economic status. It is known that LDCT is effective due to the detection of lung cancer at an earlier stage, allowing more effective treatments including surgery.

It is an important finding that at long term follow up there was no excess incidence of lung cancer in the LDCT arm. This suggests that there is little or no overdiagnosis associated with LDCT screening. Since UKLS had a single screen design, one might have expected cancer incidence rates to converge sooner than they did. This may be affected by a greater awareness of lung cancer as a result of the intervention in the screened population, or more directly by participants being kept on long term follow-up for nodules detected during LDCT. One way to investigate that is to study the stage distribution of cancers detected subsequent to the LDCT screen, i.e. excluding the 42 screen-detected cancers. Even after screening ceased, there were more early-stage cancers detected in the LDCT group, irrespective of IMD group, contributing to equivalent outcomes following diagnosis.

Nevertheless, it was interesting to note that convergence for the upper quintiles was delayed compared to the lower quintiles by approximately two years. This result might be due to greater awareness, in the generally better educated upper quintiles, augmented by the intervention leading to better medical engagement; or due to greater access to healthcare. From a policy perspective, it may suggest that those in lower quintiles require more frequent awareness or screening interventions and more help in access (e.g. local availability of LDCT facilities).

The overall improvement in lung cancer outcome for the CT arm is largely due to screen detected lung cancers, but these results indicate that there are also longer-term gains in terms of stage reduction that contribute to longer term improved outcomes, regardless of IMD grouping. Given that outcomes from time of diagnosis were the same for lower and upper quintiles, with similar proportions of early-stage disease, the impact of LDCT on all-cause mortality following screening is more likely related to the incidence of lung cancer and of competing comorbidities, both of which are more prevalent in lower quintiles.

Whilst the UKLS is not powered to detect LDCT related differences in mortality, the difference between the IMD groups is striking and indicates that there may be significantly more benefit in the lower socioeconomic groups. This requires further examination in other studies, but is likely due to the higher incidence of lung cancer and other smoking-related diseases in the lower IMD quintiles.

Given the pattern seen for lung cancer specific mortality (Fig. 3B), the apparent benefit of LDCT screening on all-cause mortality in the lower IMD quintiles (Fig. 3A) is unlikely to be due to lung cancer prior to five years; it may be that detection of other conditions (e.g. cardiac disease or COPD, which are more prevalent in this group) is a significant factor in improved health outcomes on all-cause mortality at earlier time-points. Meanwhile, these data sound a warning to implementation studies focussing on deprived areas, in terms of the expectation for early benefits in lung cancer specific mortality.

Risk-based selection for lung cancer screening has some potential benefits in terms of health equity, as the focus of the screening activity is biased to the more deprived areas, where lung cancer risk is greatest. Hence even though those in upper quintiles were more likely to be risk questionnaire respondents, the high-risk group, invited to LDCT targeted lung health checks, are more likely to be from the lowest IMD quintile. Unlike less-selective screening programmes, we therefore do not expect a detrimental impact on health inequalities, given risk-based interventions help provide some equity by selecting those most at need. We should not however be complacent, if we can improve engagement in the lower quintiles, the benefits of screening will be even further enhanced.^10^ That this socially deprived group required additional effort to fully address their health needs is exemplified by their relatively low response rate and it is likely due in part to the degree of fatalism and other negative attitudes in this community, requiring better approaches to screening communication.^15^

Risk-based screening, whether through enrichment for heavy smokers, or the contribution from prior lung disease and other cancer history, increases the proportion of the screened population that have comorbidities, such as COPD. Such comorbidities can impact the effectiveness of screening when considering overall mortality benefit, although this was found not to be the case for COPD or stroke in the PLCO trial.^16^ Nevertheless, the benefits and harms of screening vary based on the risk of lung cancer and the risk of death from competing causes has been a cause of concern.^17^ However, our data shows that, at least for the more deprived portion of society, additional benefits in terms of reduced death from COPD, and potentially other smoking-related diseases, might offset the confounding reduction in benefit due to enrichment of competing causes of death in the high-risk cohort.

In this exploratory analysis, LDCT screening apparently had a significant impact on the rates of death from other smoking-related diseases, notably COPD and emphysema in the more deprived IMD quintiles. It should be noted that spirometry was performed as part of UKLS for both LDCT and control groups, so the benefit seen may be attributed to participation in the LDCT arm, though not necessarily the LDCT itself given the possibility of unmeasured confounding factors. In the case of cardiovascular disease, coronary artery calcification reporting was not part of UKLS, but radiographers were free to report it (data unavailable for number of cardiac referrals). There was some evidence that the gains in reduced deaths due to cardiovascular disease were greater in the lower IMD quintile group, with greater divergence of the cumulative hazard curves, but this did not reach statistical significance. The impact of LDCT on these and other smoking-related diseases may be related to improved smoking cessation (data unavailable), improved medical awareness or willingness to engage. However, we have no clinical evidence that it was possibly due to improved diagnosis (especially of COPD). Nevertheless, that the effects of LDCT screening on other diseases were greater in the more deprived quintiles may partly explain why all cause mortality is lower in the more deprived LDCT group, but not the less deprived LDCT group.

The UKLS study, although a randomised control study, was of limited size, as it was only funded as a pilot study. Nevertheless, significant results for several key lung cancer outcome measures were achieved. Whilst a significant effect on time to COPD-related death was also observed, it is possible that other non-significant trends, were the result of the study being underpowered for these post-hoc analyses. It should be noted that some of the interesting outcome observations occur after 6 years and there is a drop-off in numbers exposed to risk by 8 years, which is a potential limitation, However, most of this drop is from 7 years onwards and does not impact greatly on the conclusions. The IMD data used was based on postcode rather than individual assessment (e.g. through questionnaire), so it is possible that there is some contamination between groups. However, the impact of this is somewhat limited by the pooling of IMD quintiles into 2 broader groups (Q1-Q2 and Q3-Q5). With a larger study, it might be possible to demonstrate differences between individual quintiles (although in Europe many early implementation studies are focussing on socially deprived areas, so this data might have to wait for wider implementation).

In the USA, national lung cancer screening means that the impact of socioeconomic status can be studied in detail.^18^^,^^19^ However, some of the social determinants in healthcare access and outcomes in the USA are different to Europe, most notably economic disadvantage and ethnicity.

It should be recognised that use of the LLP lung cancer risk model, to select those for recruitment, limits the finding to this high-risk group, which is naturally biased towards older ages with greater smoking history (although with some differences in different IMD quintiles). However, as this is the group for which lung cancer screening is proposed, this is not a significant limitation. Whilst smoking cessation advice and spirometry was provided to both arms of the study, irrespective of LDCT provision, follow-up data to assess the effectiveness of these interventions across different socioeconomic groups is limited. Similarly, direct data on the impact of COPD diagnosis instigated by the UKLS LDCT scan is not available, since it is unclear if primary care physicians acted on the CT reports provided. Nevertheless, that a benefit was seen in the LCDT arm suggests that the LDCT element had some direct impact, over and above trial-based spirometry and smoking awareness.

Future research is required to confirm these improved long-term outcomes for those who received a LDCT scan and to investigate the reasons behind them. In addition to improved diagnosis via LDCT (for COPD and cardiovascular disease alongside lung cancer), this might include being more responsive to smoking cessation, or better motivated to seek medical help. It would be helpful if data on other disease referrals, diagnoses and outcomes is collated systematically alongside implementation of LDCT screening.

In conclusion, this post-hoc analysis, provides novel insights on the influence of socioeconomic status on LDCT screening in a European setting. Although lung cancer screening effectiveness did not differ between lower and higher socioeconomic groups, a lower participation rate was seen in the most deprived quintile. However, utilisation of risk-based selection effectively helped restore equity to LDCT provision. Furthermore, outcome from lung cancer was as good for the more deprived quintiles (IMD Q1, Q2) as for the less deprived (IMD Q3, Q4, Q5) in this lung cancer screening, early diagnosis setting; with both groups having similar benefits in terms of early stage disease. In addition, there was a significant, unexplained reduction on the rates of death from other smoking-related diseases, notably COPD and emphysema, that was seen exclusively in lower socioeconomic groups.

## Contributors

MPAD: Conceptualization, Methodology, Formal analysis, Data Curation, Writing–Original Draft, Writing–Review & Editing, Project administration. DV: Methodology, Formal analysis, Writing–Review & Editing, Visualization. RG: Conceptualization, Writing–Review & Editing, Supervision. SWD: Conceptualization, Methodology, Writing–Review. & Editing, Supervision, Funding acquisition. JKF: Conceptualization, Resources, Writing–Review & Editing, Supervision, Project administration, Funding acquisition. All authors reviewed and approved the manuscript.

## Data sharing statement

Individual participant data that underlie the results reported in this article, after de-identification (text, tables, figures, and appendices) may be made available upon request to researchers who provide a methodologically sound proposal. Proposals should be directed to J.K.Field@liverpool.ac.uk; to gain access, data requestors will need to sign a data access agreement. Analyses will be limited to those approved in appropriate ethics and governance arrangements. All study documents which do not identify individuals (e.g. study protocol, statistical analysis plan, informed consent form) will be freely available on request.

## Declaration of interests

JKF has received fees from AstraZeneca (Speaker's Bureau) and advisory boards of Epigenomics; NUCLEIX Ltd. AstraZeneca, iDNA; Grant Support: Janssen Research & Development, LLC. No competing interests from all other co-authors.

## References

[1] UK Ministry of Housing CLG (2011). https://assets.publishing.service.gov.uk/government/uploads/system/uploads/attachment_data/file/6871/1871208.pdf.

[2] Payne N.W.S., Brown K.F., Delon C., Kotrotsios Y., Soerjomataram I., Shelton J. (2022). Socio-economic deprivation and cancer incidence in England: quantifying the role of smoking. PLoS One.

[3] UK CR (2020). Cancer in the UK 2020: socio-economic deprivation. https://www.cancerresearchuk.org/sites/default/files/cancer_inequalities_in_the_uk.pdf.

[4] Statistics. OfN (2023). Deprivation and the impact on smoking prevalence, England and Wales: 2017 to 2021. https://www.ons.gov.uk/peoplepopulationandcommunity/healthandsocialcare/drugusealcoholandsmoking/bulletins/deprivationandtheimpactonsmokingprevalenceenglandandwales/2017to2021.

[5] Hovanec J., Siemiatycki J., Conway D.I. (2018). Lung cancer and socioeconomic status in a pooled analysis of case-control studies. PLoS One.

[6] Finke I., Behrens G., Weisser L., Brenner H., Jansen L. (2018). Socioeconomic differences and lung cancer survival-systematic review and meta-analysis. Front Oncol.

[7] Aberle D.R., Adams A.M., National Lung Screening Trial Research T (2011). Reduced lung-cancer mortality with low-dose computed tomographic screening. N Engl J Med.

[8] de Koning H.J., van der Aalst C.M., de Jong P.A. (2020). Reduced lung-cancer mortality with volume CT screening in a randomized trial. N Engl J Med.

[9] Field J.K., Vulkan D., Davies M.P.A. (2021). Lung cancer mortality reduction by LDCT screening: UKLS randomised trial results and international meta-analysis. Lancet Reg Health Eur.

[10] Oudkerk M., Liu S., Heuvelmans M.A., Walter J.E., Field J.K. (2021). Lung cancer LDCT screening and mortality reduction–evidence, pitfalls and future perspectives. Nat Rev Clin Oncol.

[11] Rollet Q., Tron L., De Mil R., Launoy G., Guillaume E. (2021). Contextual factors associated with cancer screening uptake: a systematic review of observational studies. Prev Med.

[12] Field J.K., Duffy S.W., Baldwin D.R. (2016). The UK Lung Cancer Screening Trial: a pilot randomised controlled trial of low-dose computed tomography screening for the early detection of lung cancer. Health Technol Assess.

[13] Field J.K., Vulkan D., Davies M.P.A., Duffy S.W., Gabe R. (2021). Liverpool Lung Project lung cancer risk stratification model: calibration and prospective validation. Thorax.

[14] Baldwin D.R., Duffy S.W., Wald N.J., Page R., Hansell D.M., Field J.K. (2011). UK Lung Screen (UKLS) nodule management protocol: modelling of a single screen randomised controlled trial of low-dose CT screening for lung cancer. Thorax.

[15] Quaife S.L., Marlow L.A.V., McEwen A., Janes S.M., Wardle J. (2017). Attitudes towards lung cancer screening in socioeconomically deprived and heavy smoking communities: informing screening communication. Health Expect.

[16] Robinson E.M., Liu B.Y., Sigel K., Yin C., Wisnivesky J., Kale M.S. (2022). Impact of comorbidities on lung cancer screening evaluation. Clin Lung Cancer.

[17] Howard D.H., Richards T.B., Bach P.B., Kegler M.C., Berg C.J. (2015). Comorbidities, smoking status, and life expectancy among individuals eligible for lung cancer screening. Cancer.

[18] Adnan S.M., Chin K., Ma G.X., Erkmen C.P. (2023). A narrative review of the social determinants of lung cancer screening: knowledge gaps and controversies. Curr Chall Thorac Surg.

[19] Haddad D.N., Sandler K.L., Henderson L.M., Rivera M.P., Aldrich M.C. (2020). Disparities in lung cancer screening: a review. Ann Am Thorac Soc.

