# Supplementary material for: Impact of single round of low dose CT lung cancer screening on cause of mortality in different socio-economic groups: a post-hoc analysis of long-term follow-up of the UKLS trial
Source: Lancet Reg Health Eur. 2024 May 20;42:100936. doi: 10.1016/j.lanepe.2024.100936 (PMC11134881; doi:10.1016/j.lanepe.2024.100936)

Impact of single round of low dose CT screening on cause of mortality in different socio-economic groups, a post-hoc analysis of long-term follow-up of the UKLS trial

Supplementary Data

CONSORT 2010 Flow Diagram

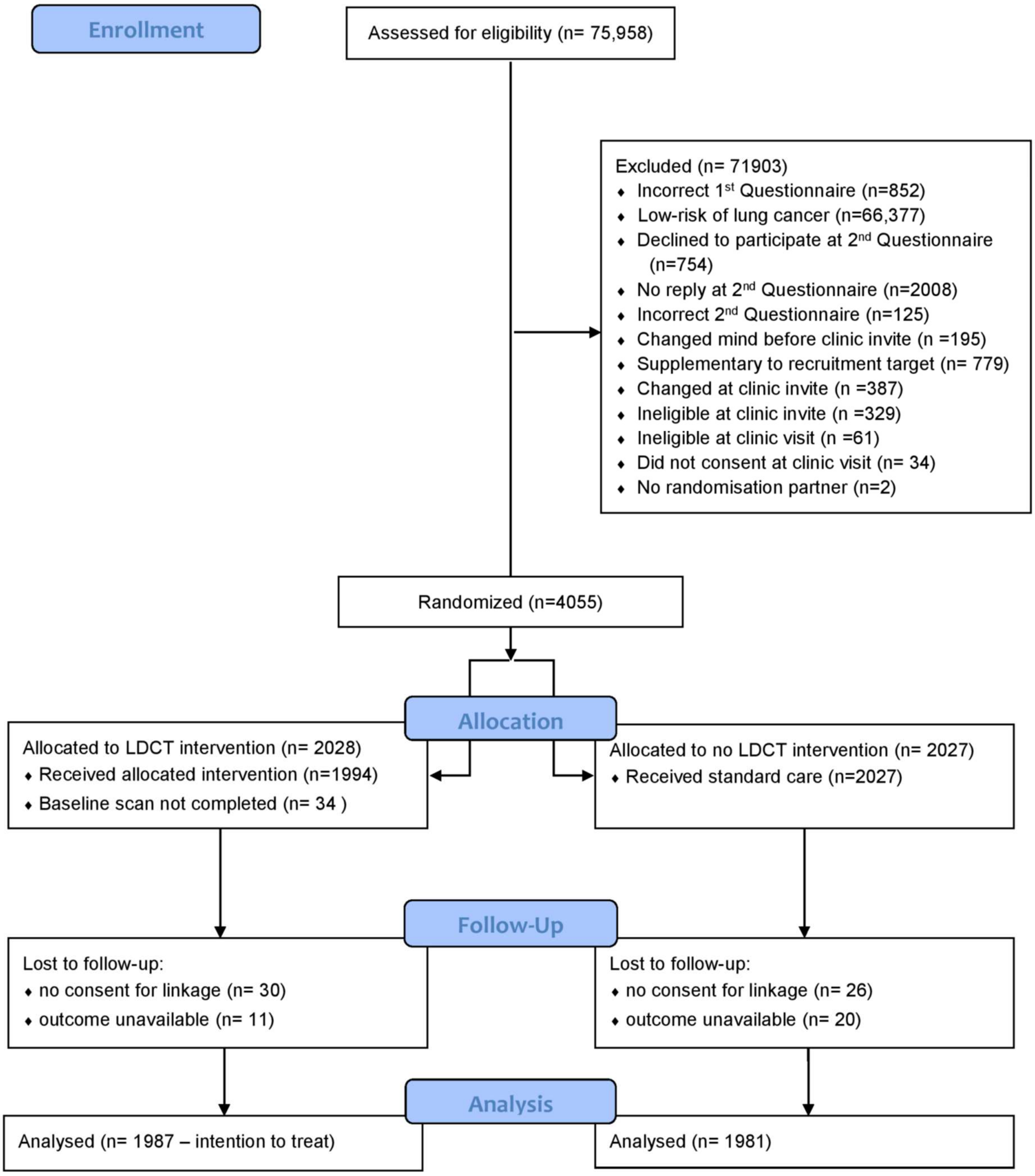

Impact of recruitment and risk selection on IMD profile and risk factor profiles

The UKLS target population consisted of two geographical areas with differing IMD profiles. Utilising the 75,948 UKLS questionnaire responders who provided adequate information both for IMD scoring and LLPv2 risk scores, we have examined the distribution of IMD quintiles. Notably the IMD profile of the total pool of respondents is skewed towards the upper quintiles, reflecting greater engagement and the higher proportion of respondents from the south (57%) which is more affluent.

As expected, compared to the total respondents, the IMD profile for the high-risk group is skewed toward the lower quintiles (chi-square P<0·001). Despite this the IMD profiles of the two trial participant groups (no CT and LDCT, representing 42.5% of the high-risk group) is less skewed than the total high-risk group, with a lower proportion of Q1 and a higher proportion of Q5, indicating that there is likely a further selective pressure against lower IMD groups for trial participation at this stage of section. Randomisation to LDCT was not biased by IMD (chi-square P = 0·91).

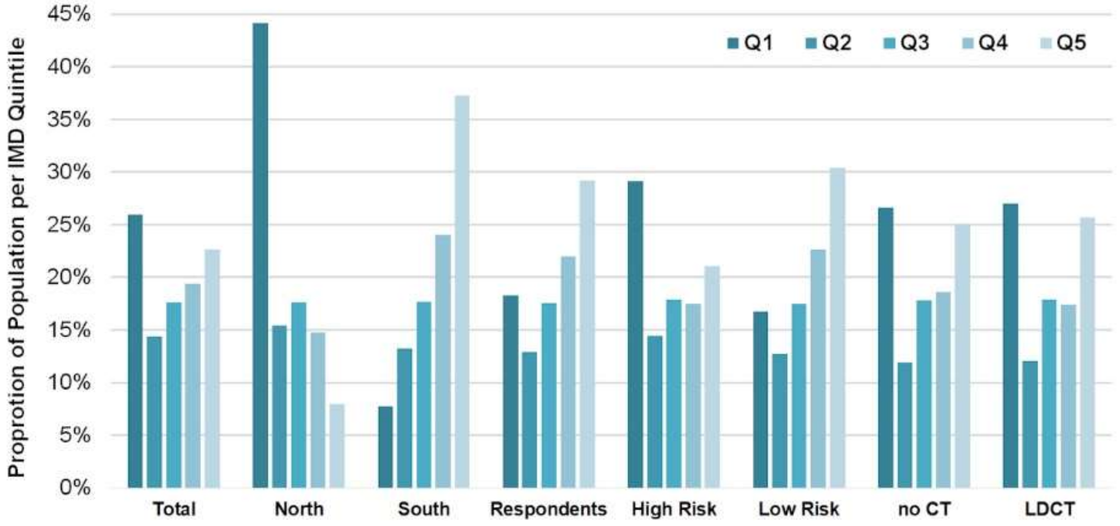

Figure S1 IMD profiles for UKLS target population and subgroups based on region, questionnaire response, risk group and LDCT treatment.

Total target population (n= 249,962), North target population (n= 124,995), South target population (n= 124,967), questionnaire responders (n= 75,498), high risk (n= 9,531), low risk (n= 66,417), no CT trial participants (n= 2061), and those receiving LDCT (n= 1994).

Relationship between individual LLP risk score variables and IMD in UKLS responders

Across the total 75,096 respondents with full risk factor data and IMD data, associations between lung cancer risk factors and lower IMD quintile were seen for all except history of pneumonia and personal history of cancer, both of which were more uniformly distributed (Table S1).

In the subgroup of 8,728 high-risk UKLS respondents (Table S2) only COPD, emphysema and family history of lung cancer were still significantly associated with lower IMD quintiles. History of asbestos exposure (previously associated with lower quintiles), pneumonia and personal history of cancer (both previously not associated with IMD) were associated with upper IMD quintiles. This represents the selection bias imposed by use of the LLP lung cancer risk score, when compared to the whole cohort, but may also be related to the greater proportion of upper quintile respondents (with generally lower smoking-related risk) for which these factors contributed significantly to their high-risk score.

In the 4,055 risk-selected trial participants (Table S3), only, COPD and family history of lung cancer were significantly associated with lower IMD quintiles, with some association for TB. History of pneumonia and personal history of cancer were both associated with upper IMD quintiles, as for the high-risk respondents in general, but asbestos exposure was no longer significant. Presumably there was some additional selection bias for those able and willing to take part in the LDCT screen, although the smaller sample size will reduce statistical significance.

Table S1 Categorical lung cancer risk factors and IMD in 75,096 UKLS respondents (red= high values, clear = middling values, blue = low values, scaled to range per variable)

IMD Quintile	Asbestos	Pneumonia	COPD	Emphysema	Bronchitis	TB	Personal history of cancer	Family history of lung cancer <60y	Family history of lung cancer >60y
Q1	16·3%	8·8%	9·7%	2·7%	16·2%	2·3%	11·2%	9·4%	13.0%
Q2	15·3%	9·3%	5·2%	1·9%	15·3%	2·0%	11·4%	5·9%	11.1%
Q3	14·8%	9·2%	4·1%	1·8%	15·0%	2·0%	11·8%	5·8%	11.1%
Q4	14·0%	9·3%	2·9%	1·3%	14·4%	1·9%	11·5%	5·0%	9.7%
Q5	13·2%	8·9%	2·1%	1·0%	13·6%	1·4%	11·4%	4·4%	9.5%
Chi-square P	10^{-15}	0·38	10^{-276}	10^{-35}	10^{-10}	10^{-08}	0·70	10^{-120}	

Table S2 Categorical lung cancer risk factors and IMD in 8,728 high-risk UKLS respondents (colours as for Table S1)

IMD Quintile	Asbestos	Pneumonia	COPD	Emphysema	Bronchitis	TB	Personal history of cancer	Family history of lung cancer <60y	Family history of lung cancer >60y
Q1	31·5%	13·9%	26·6%	6·9%	27·2%	4·0%	18·7%	15·7%	14.3%
Q2	33·0%	19·5%	19·7%	7·0%	28·7%	3·1%	18·0%	11·8%	15.3%
Q3	34·1%	18·1%	16·2%	7·1%	29·2%	4·2%	22·1%	11·6%	13.4%
Q4	36·5%	18·6%	11·7%	5·3%	26·7%	3·4%	20·0%	10·8%	13.0%
Q5	34·9%	17·6%	10·8%	4·8%	26·7%	3·2%	22·4%	9·1%	10·5%
Chi-square P	0·015	0·00002	10^{-50}	0·007	0·363	0·364	0·002	10^{-13}	

Table S3 Categorical lung cancer risk factors and IMD in 4,055 UKLS trial subjects (colours as for Table S1)

IMD Quintile	Asbestos	Pneumonia	COPD	Emphysema	Bronchitis	TB	Personal history of cancer	Family history of lung cancer <60y	Family history of lung cancer >60y
Q1	38·1%	13·5%	25·0%	5·3%	28·5%	4·5%	17·3%	17·5%	15.5%
Q2	36·2%	18·1%	15·6%	5·3%	30·2%	2·3%	17·1%	10·7%	18.1%
Q3	37·2%	19·6%	15·2%	6·4%	30·8%	4·6%	23·4%	9·8%	14.7%
Q4	38·1%	21·0%	10·5%	5·2%	26·0%	2·7%	19·9%	10·8%	13.4%
Q5	38·5%	17·3%	10·0%	4·0%	27·1%	3·0%	21·8%	8·6%	10.8%
Chi-square P	0·93	0·0004	10^{-23}	0·27	0·21	0·046	0·0052	10^{-12}	

In the case of age, compared to all respondents (Figure S2A), age was higher in upper IMD quintiles in the high-risk group (Figure S2B). Whilst there was an even balance of gender in the total respondent group across all IMD quintiles (Figure S2C), the risk score significantly favours inclusions of males, but less so in the lowest quintile (Figure S2D). Greater smoking duration (Figure S2E & F) was clearly associated with lower IMD, with risk selection, as expected the higher smoking duration groups were selected, but the main difference between IMD quintiles was in the 40-60 year smoking group.

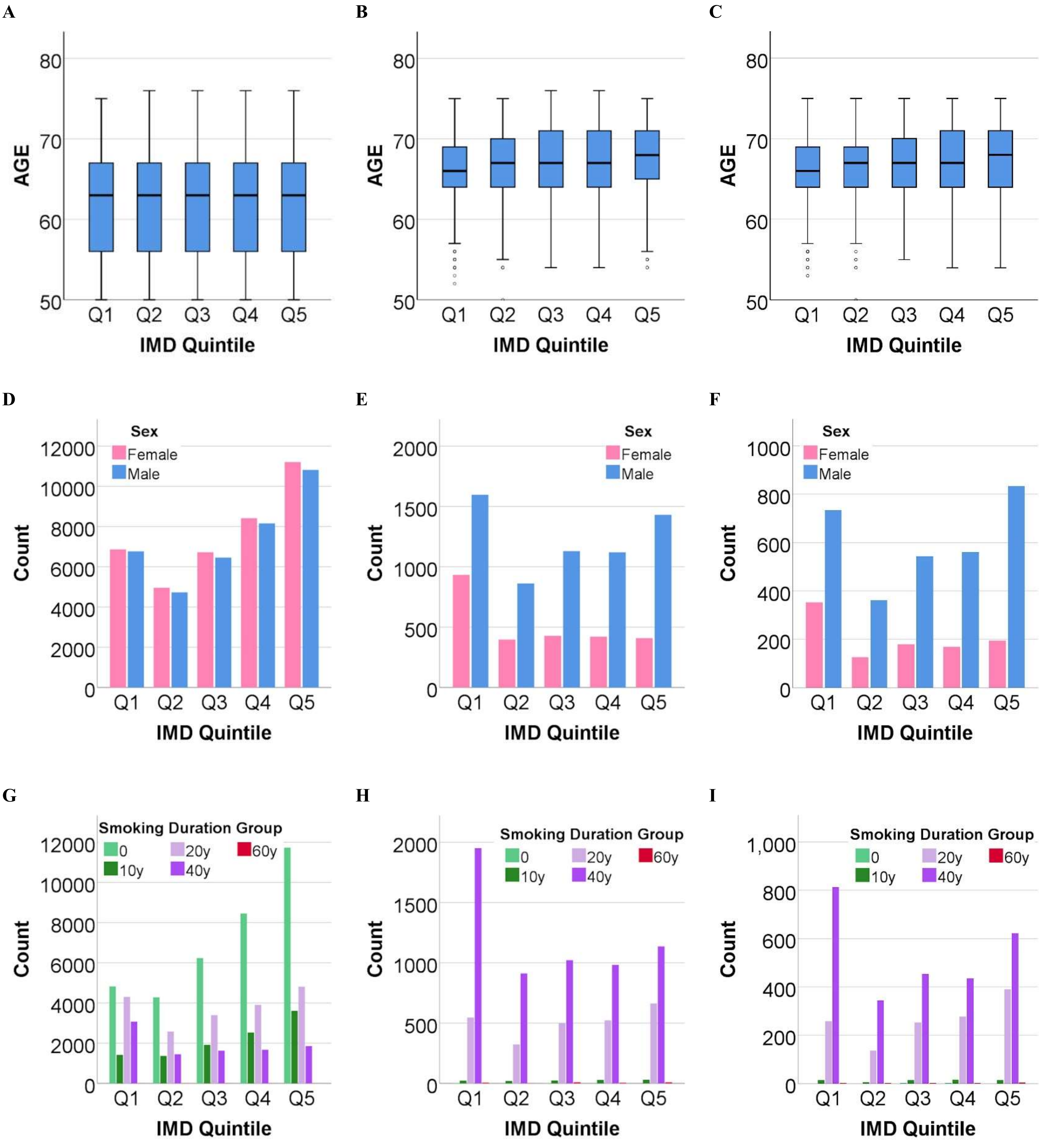

Figure S2 Age, sex and smoking duration by IMD group, effect of risk selection

A - Age all respondents (Kruskal-Wallis P< 0·001), B - Age high-risk (Kruskal-Wallis P<0·001), C – Age trial cohort (Kruskal-Wallis P<0·001) D - gender all respondents (chi-square P= 0·77), E - gender high-risk (chi-square P< 0·001), F - gender trial cohort (chi-square P< 0·001), G - smoking all respondents (Kruskal-Wallis P< 0·001), H - smoking high-risk (Kruskal-Wallis P< 0·001), I - smoking trial cohort (Kruskal-Wallis P< 0·001).

Association of risk factors with IMD quintile in the trial population

A

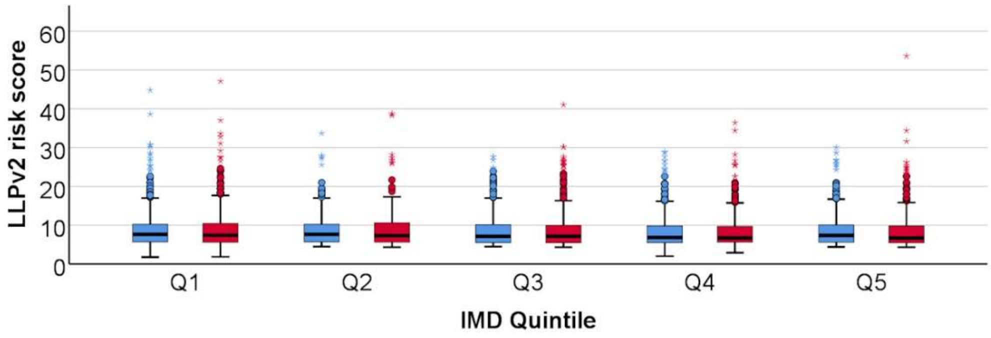

B

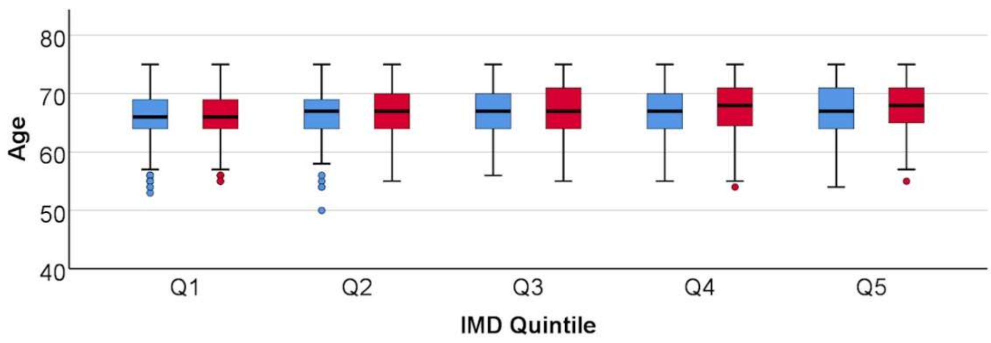

C

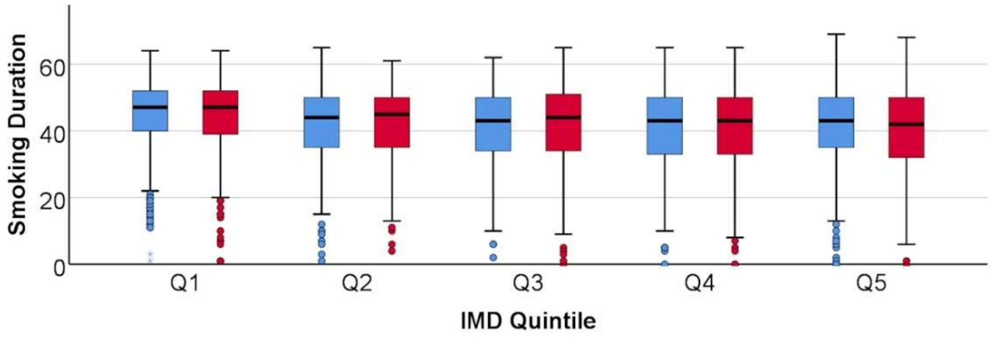

Figure S3 Risk score (A), age (B) and all smoking duration (C) variation between IMD quintiles in LDCT (red) and usual care (blue) cohorts. Further details for IMD quintiles are provided in Table 1.

Relationship of IMD with LLP risk score, lung cancer incidence and mortality in the absence of LDCT screening

Utilising the 75,096 UKLS responders, but excluding those 1994 participants that received LDCT, the 5-year incidence of lung cancer (Figure S4) follows the expected relationship with IMD in both the high-risk group (LLPv2 >4.5%) eligible for LDCT and the remaining low risk group.

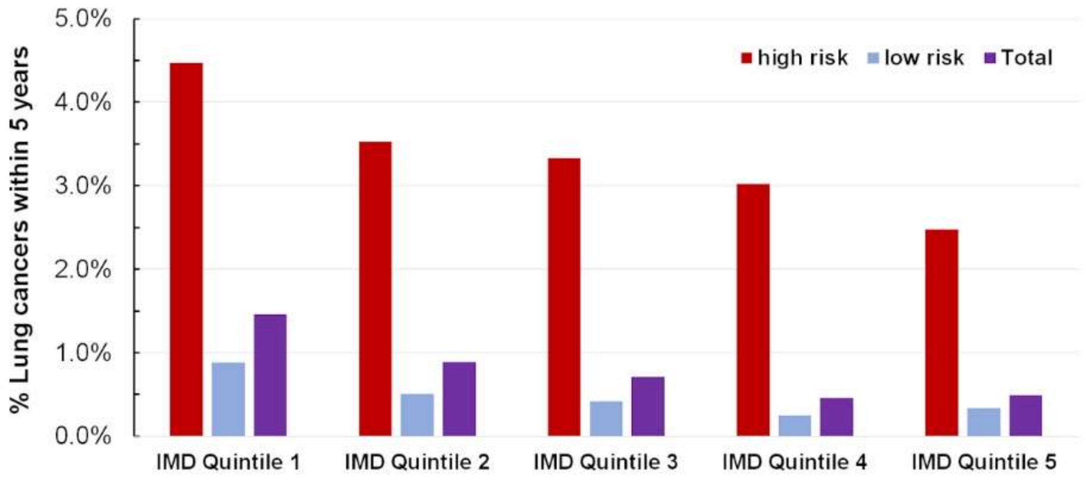

Figure S4 5-year lung cancer incidence across IMD quintiles for subgroups of UKLS questionnaire responders. High risk (LLPv2 >4.5%, n= 6734) and low risk group (n=66368).

There appears to be selection bias in terms of lung cancer incidence, introduced when looking at UKLS participants (excluding those who received LDCT), with quintile 1 having a lower incidence than might be expected and quintile 5 a high incidence (Figure S5A). Notably Q2 initially had a greater incidence of lung cancer and higher mortality than Q1 and Q5 a greater incidence than either Q3 or Q4, although these differences are not statistically significant. The same is true for lung cancer mortality (Figure S5B).

Whilst differences in lung cancer risk factors may contribute towards the observed lung cancer incidence and outcome patterns between IMD quintiles, they were narrower within this age and risk-score selected cohort than in the general population.

A more useful analysis of lung cancer incidence and outcome can be achieved by grouping the lower two (Q1-2) and the upper three (Q3-5) IMD quintiles (Figure S6).

A

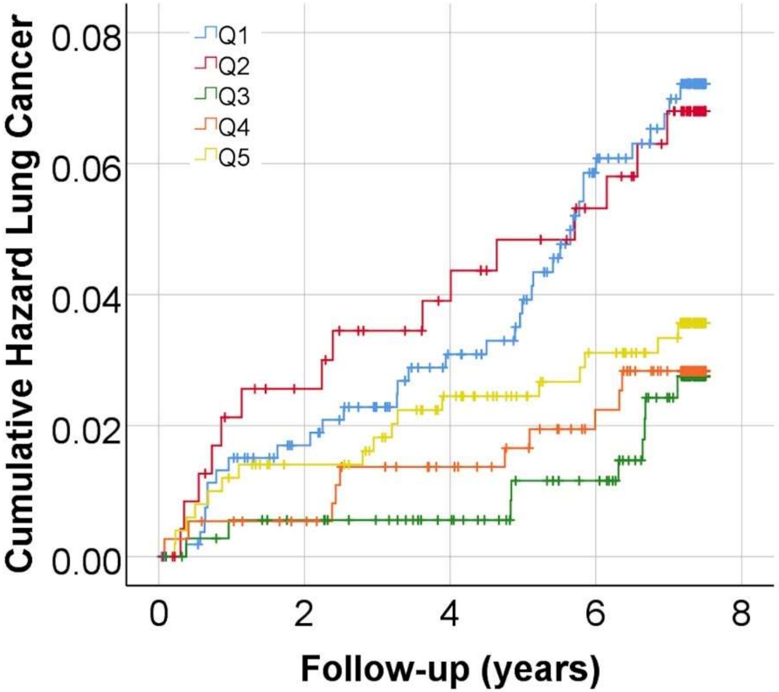

Quintile	Number Exposed to Risk (Events)			
1	532 (9)	507 (7)	478 (13)	280(6)
2	237 (6)	224 (3)	213 (3)	131 (3)
3	359 (2)	348 (0)	336 (2)	201 (6)
4	370 (2)	361 (3)	347 (3)	205 (2)
5	498 (7)	481 (5)	462 (3)	266 (2)

B

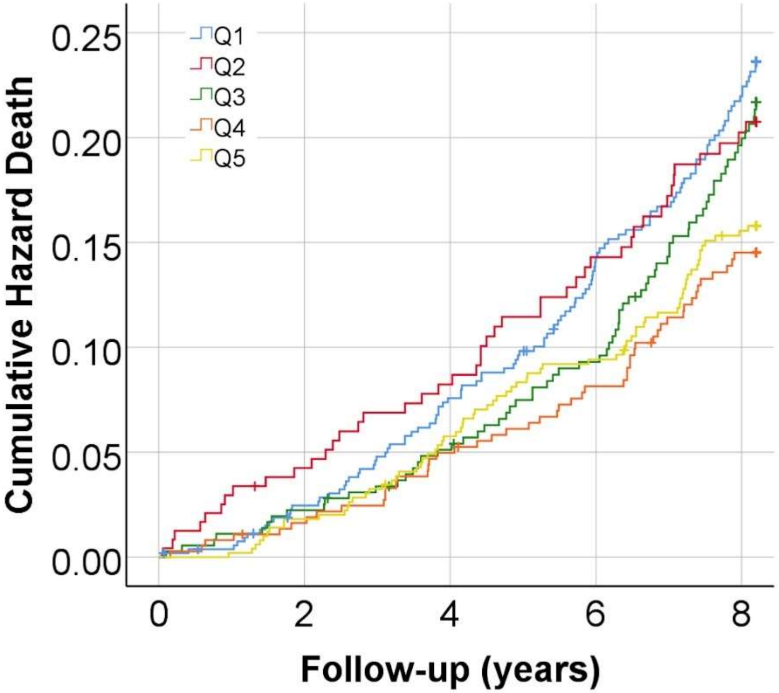

Quintile	Number Exposed to Risk (Events)				
1	536 (13)	521 (26)	494 (31)	461 (35)	226 (26)
2	241 (10)	230 (9)	221 (13)	208 (12)	103 (10)
3	362 (8)	353 (10)	342 (14)	327 (32)	155 (16)
4	372 (6)	365 (12)	353 (11)	341 (21)	163 (6)
5	501 (9)	492 (19)	472 (17)	454 (27)	221 (16)

Figure S5 Lung cancer incidence (A, log rank P = 0·004) and all-cause mortality (B, log rank P = 0·015) in the UKLS control cohort by IMD quintile (Q1 – Q5). The number exposed at time points 0, 2, 4 and 6years (A) or 0, 2, 4, 6 and 8 years (B) are given, with the number of events in the subsequent time period (in brackets).

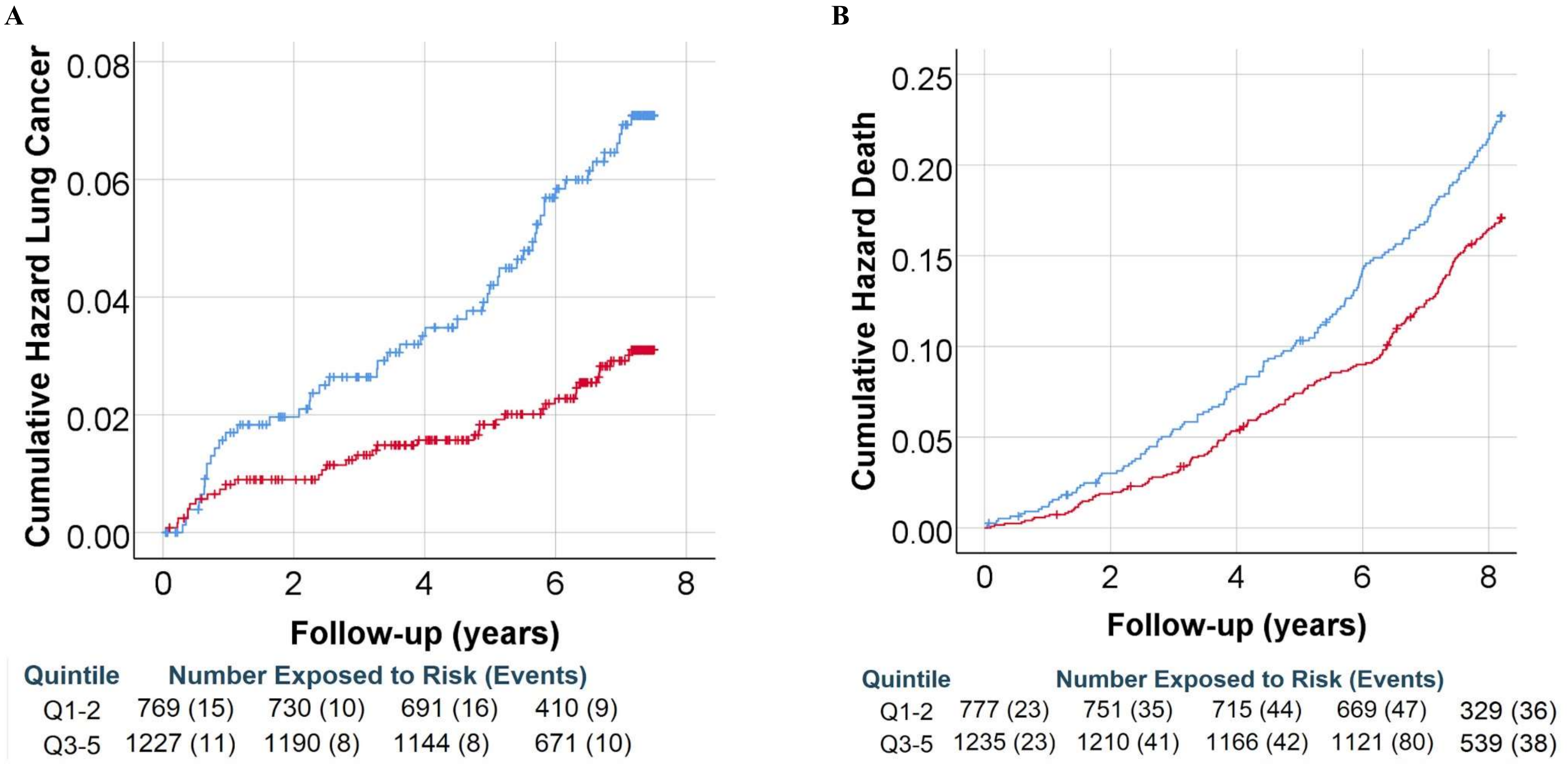

Figure S6 Lung cancer incidence (A, log rank P < 0·001) and all-cause mortality (B, log rank P = 0·0045) in UKLS control cohort grouped as more deprived quintiles Q1-Q2 (blue) vs less deprived quintiles Q3-Q5 (red). The number exposed at time points 0, 2, 4 and 6years (A) or 0, 2, 4, 6 and 8 years (B) are given, with the number of events in the subsequent time period (in brackets).

Relationship of IMD with histological type

Looking at histological type distributions in different IMD quintiles, as expected there was a higher proportion of squamous cell carcinoma in the most deprived quintile (Q1 35.6%) than in other quintiles (Q2 11.5%, Q3 11.5%, Q4, 15.8%, Q5 16.7%, chi-square Q1 vs Q2-4 P= 0.005), potentially associated with greater smoking in the most deprived quintile, given squamous cell carcinoma has a greater association with smoking.

However, when looking at the quintile groups used for outcome analysis, whilst the proportion of squamous cell carcinoma was still higher in the more deprived group (Q1-2 29.3%) compared to the less deprived (Q3-5 14.7%), the distribution of histological type within these two IMD groups was not significantly different when looking at all histological types (chi-square P = 0.57) and only borderline when looking at the ratio of adenocarcinoma to squamous cell carcinoma (chi-square P = 0.041).

Similar patterns were seen in both arms of the trial, i.e. whether or not the subjects received LDCT.

Table S4 Distribution of histological type by IMD quintile

Histology		IMD Quintile					Total
		Q1	Q2	Q3	Q4	Q5	
AdC	Count	31	12	15	8	15	81
	% within quintile	42.5%	46.2%	57.7%	42.1%	50.0%	46.6%
SqC	Count	26	3	3	3	5	40
	% within quintile	35.6%	11.5%	11.5%	15.8%	16.7%	23.0%
NOS	Count	6	5	6	2	4	23
	% within quintile	8.2%	19.2%	23.1%	10.5%	13.3%	13.2%
SCLC	Count	6	4	2	1	4	17
	% within quintile	8.2%	15.4%	7.7%	5.3%	13.3%	9.8%
NSCLC	Count	4	1	0	1	0	6
	% within quintile	5.5%	3.8%	0.0%	5.3%	0.0%	3.4%
Carcinoid	Count	0	1	0	3	2	6
	% within quintile	0.0%	3.8%	0.0%	15.8%	6.7%	3.4%
Large Cell	Count	0	0	0	1	0	1
	% within quintile	0.0%	0.0%	0.0%	5.3%	0.0%	0.6%
	Total	73	26	26	19	30	174

Adjustment of IMD-related outcomes for other risk factors

The UKLS statistical Analysis Plan states that no adjustment to outcome analysis is made for risk factors that are balanced between trial arms and all risk factors were well balanced. However the same is not true for analysis of IMD. We have demonstrated, in keeping with known demographic trends, that the more deprived (Q1-2) group is associated with greater smoking duration. However, they also tended to be younger (as those in the less deprived quintiles had to be older to achieve the same risk score, given lower smoking duration). Whilst statistically significant, these differences are very small in scale, most probably as a result of all participants being in the high-risk category. Nevertheless, we have checked the Cox regression models by adjusting for age and smoking duration.

Table S5 Cox regression analysis by IMD group, age and smoking duration

Outcome	Cox model	factor	HR	95% CI	p
all-cause mortality	unadjusted	Q1-2 vs Q3-5	0.818	0.717 – 0.933	0.003
	adjusted	Q1-2 vs Q3-5	0.781	0.684 – 0.892	<0.001
		Smoking duration	1.012	1.006 – 1.017	<0.001
		Age	1.097	1.078 – 1.116	<0.001
lung cancer mortality	unadjusted	Q1-2 vs Q3-5	0.447	0.311 – 0.641	<0.001
	adjusted	Q1-2 vs Q3-5	0.480	0.333 – 0.691	<0.001
		Smoking duration	1.049	1.029 – 1.069	<0.001
		Age	1.038	0.990 – 1.087	0.12
COPD/Emphysema	unadjusted	Q1-2 vs Q3-5	0.580	0.352 – 0.893	0.015
	adjusted	Q1-2 vs Q3-5	0.567	0.354 – 0.909	0.018
		Smoking duration	1.044	1.020 – 1.069	<0.001
		Age	1.102	1.035 – 1.174	0.003

Figure S7 Cause of death in groups defined by IMD and LDCT (A) as % of subjects at risk with % change compared to no CT control, (B) COPD and emphysema specific mortality, stratified by LDCT and IMD, and C cardiovascular disease stratified by LDCT and IMD.

LDCT and IMD groups: Q1-2_CT = LDCT more deprived (red); Q1-2_C = Control more deprived (blue dotted); Q3-5_CT = LDCT less deprived (orange) Q3-5_C = Control less deprived (green dotted). Subjects per group in A: Q1-2_C= 780, Q1-2_CT= 760, Q3-5_C= 1236, Q3-5_CT=1194, (P= 0.005). In B and C, the number exposed at time points 0, 2, 4, 6 and 8 years (B) are given, with the number of events in the subsequent time period (in brackets).

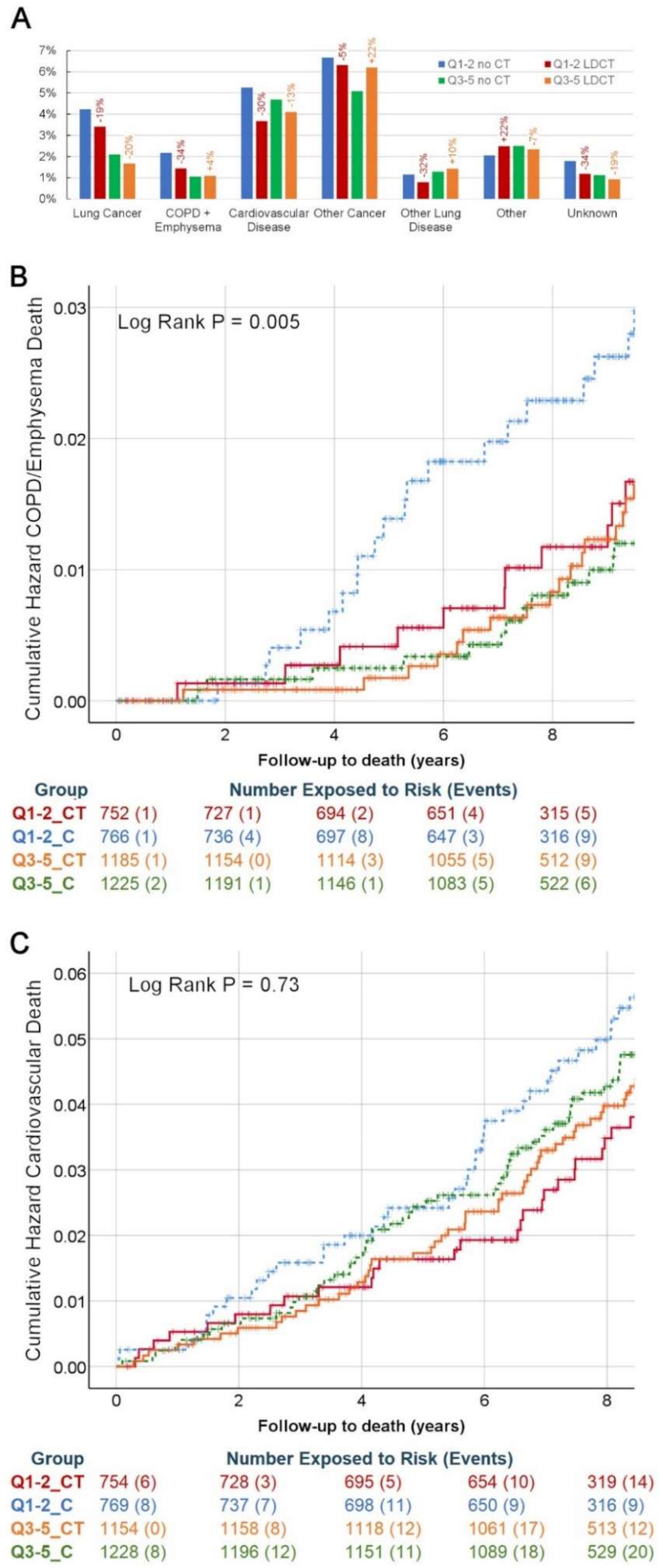

Supplement: Supplementary Figures and Tables [file mmc1.pdf]
